# Synthesis, structure and reactivity of a terminal magnesium fluoride compound, [Tp^Bu^t^,Me^]MgF: hydrogen bonding, halogen bonding and C–F bond formation†
†Electronic supplementary information (ESI) available: Experimental details. CCDC 1424708, 1424709, 1424759 and 1424773. For ESI and crystallographic data in CIF or other electronic format see DOI: 10.1039/c5sc03504j


**DOI:** 10.1039/c5sc03504j

**Published:** 2015-11-17

**Authors:** Michael Rauch, Serge Ruccolo, John Paul Mester, Yi Rong, Gerard Parkin

**Affiliations:** a Department of Chemistry , Columbia University , New York , New York 10027 , USA . Email: parkin@columbia.edu

## Abstract

The bulky tris(3-*tert*-butyl-5-pyrazolyl)hydroborato ligand, [Tp^Bu^t^,Me^], has been employed to obtain the first structurally characterized example of a molecular magnesium compound that features a terminal fluoride ligand, namely [Tp^Bu^t^,Me^]MgF, *via* the reaction of [Tp^Bu^t^,Me^]MgMe with Me_3_SnF. The chloride, bromide and iodide complexes, [Tp^Bu^t^,Me^]MgX (X = Cl, Br, I), can also be obtained by an analogous method using Me_3_SnX. The molecular structures of the complete series of halide derivatives, [Tp^Bu^t^,Me^]MgX (X = F, Cl, Br, I) have been determined by X-ray diffraction. In each case, the Mg–X bond lengths are shorter than the sum of the covalent radii, thereby indicating that there is a significant ionic component to the bonding, in agreement with density functional theory calculations. The fluoride ligand of [Tp^Bu^t^,Me^]MgF undergoes halide exchange with Me_3_SiX (X = Cl, Br, I) to afford [Tp^Bu^t^,Me^]MgX and Me_3_SiF. The other halide derivatives [Tp^Bu^t^,Me^]MgX undergo similar exchange reactions, but the thermodynamic driving forces are much smaller than those involving fluoride transfer, a manifestation of the often discussed silaphilicity of fluorine. In accord with the highly polarized Mg–F bond, the fluoride ligand of [Tp^Bu^t^,Me^]MgF is capable of serving as a hydrogen bond and halogen bond acceptor, such that it forms adducts with indole and C_6_F_5_I. [Tp^Bu^t^,Me^]MgF also reacts with Ph_3_CCl to afford Ph_3_CF, thereby demonstrating that [Tp^Bu^t^,Me^]MgF may be used to form C–F bonds.

## Abstract 2

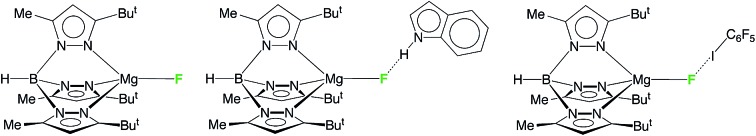
The terminal magnesium fluoride compound, [Tp^Bu^t^,Me^]MgF, serves as a hydrogen bond and halogen bond acceptor for indole and C_6_F_5_I, and also reacts with Ph_3_CCl to form a C–F bond.

## Introduction

As a consequence of its small size, high electronegativity and low polarisability, the chemistry of fluorine is often distinctly different from that of the other halogens.^1^–^4^ For example, metal fluoride compounds often exhibit novel structures^5^ and reactivity,1b,^2^,^6^,^7^,^8^,^9^ but are generally more difficult to obtain than the other halide derivatives. As an illustration, while Grignard reagents (RMgX) are readily synthesized upon treatment of magnesium with RCl, RBr, or RI, the corresponding fluoro Grignard reagents are notoriously difficult to obtain^2^–^4^ and have been investigated to a negligible extent by comparison to the other halogen derivatives. The paucity of magnesium fluoride compounds is not, however, restricted to Grignard reagents, as illustrated by the fact that fluoride derivatives comprise only 2.4% of all structurally characterized magnesium halide compounds listed in the Cambridge Structural Database (CSD).^10^ Even more striking, in none of these compounds does fluorine serve the role of a terminal ligand. Magnesium fluoride compounds are, nevertheless, of considerable importance in view of the role that they have played in biological systems. For example, the use of *in situ* generated [MgF_3_]^–^ to provide transition state analogues of the [PO_3_]^–^ moiety has generated information concerned with the mechanism of phosphoryl transfer as catalyzed by enzymes.^11^–^13^ Here, we report the synthesis and structural characterization of a terminal magnesium fluoride complex, together with its ability to participate in (i) hydrogen bonding and halogen bonding interactions (both of which are important with respect to crystal engineering),^14^ and also (ii) C–F bond formation, which is of note because of the significance of introducing fluorine into organic molecules.1a,^15^

## Results and discussion

We have previously employed tris(pyrazolyl)hydroborato ligands, [Tp^R,R′^],^16^ that feature bulky *tert*-butyl substituents, namely [Tp^Bu^t^^], [Tp^Bu^t^,Me^] and [
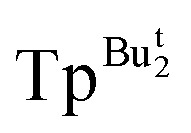
], to provide a sterically demanding pocket about a metal center that enables the isolation of a variety of novel compounds. For example, [Tp^Bu^t^,R′^] ligands provided the first structurally characterized examples of (i) a monomeric zinc hydride compound, [Tp^Bu^t^^]ZnH,^17^ (ii) a monomeric terminal zinc hydroxide compound, [Tp^Bu^t^,Me^]ZnOH,^18^ and (iii) a monomeric monovalent gallium compound, [
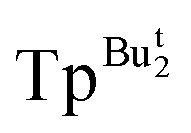
]Ga.^19^ This class of ligand was also used to synthesize monomeric magnesium chloride, bromide and iodide compounds,^20^,^21^ thereby suggesting the possibility that it could also afford a terminal magnesium fluoride compound.

**Scheme 1:**
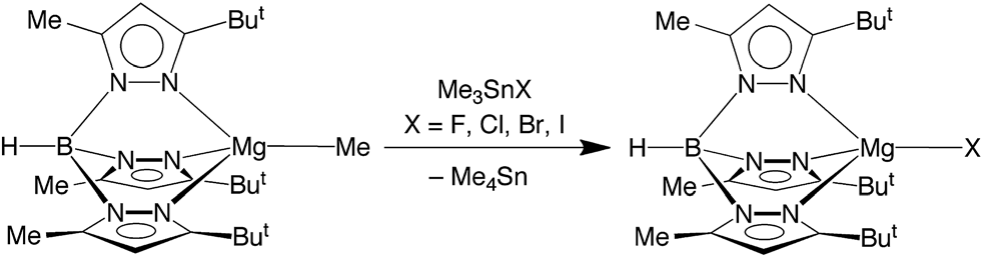
Synthesis of [Tp^Bu^t^,Me^]MgX.

Significantly, we have discovered that the fluoride compound [Tp^Bu^t^,Me^]MgF can indeed be obtained readily upon treatment of [Tp^Bu^t^,Me^]MgMe^22^ in benzene with the tin reagent, Me_3_SnF,^23^ as illustrated in Scheme 1. The chloride, bromide and iodide complexes, [Tp^Bu^t^,Me^]MgX can also be obtained by the analogous method using Me_3_SnX (X = Cl, Br, I; Scheme 1).

**Fig. 1:**
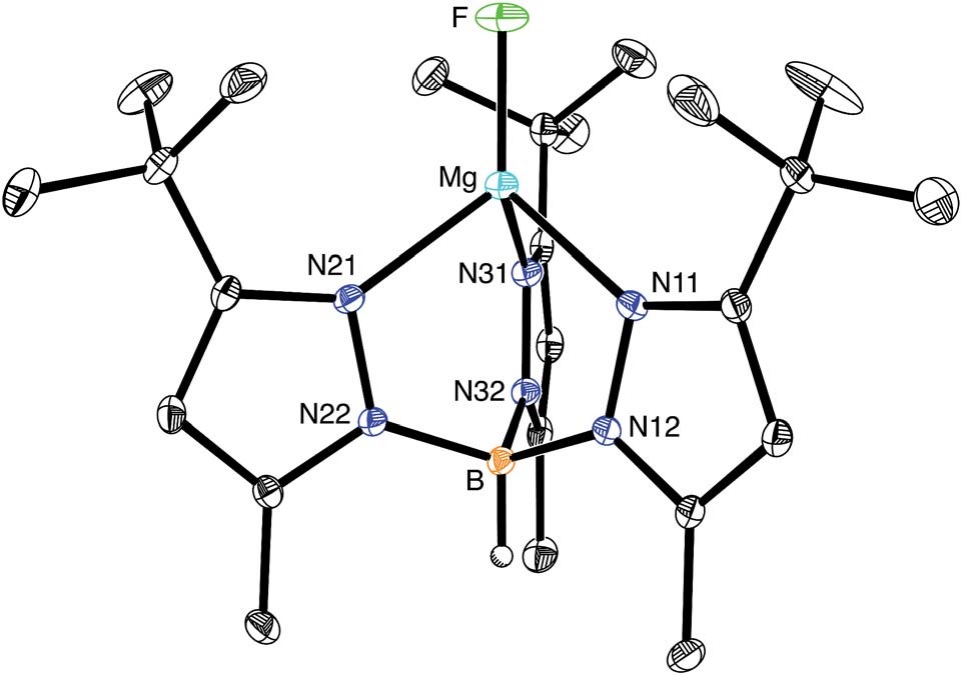
Molecular structure of [Tp^Bu^t^,Me^]MgF.

The molecular structure of [Tp^Bu^t^,Me^]MgF has been determined by X-ray diffraction (Fig. 1), thereby demonstrating that the compound is mononuclear and possesses a terminal fluoride ligand. As noted above, there are no similar compounds listed in the CSD, with other magnesium fluoride derivatives exhibiting various types of bridging interactions, which include μ_2_-,^24^–^26^ μ_3_-,^27^ and μ_4_-modes.^28^,^29^ Terminal Mg–F moieties have, nevertheless, been structurally characterized in protein structures.11a,d,e,h,i

As would be expected, the Mg–F bond of [Tp^Bu^t^,Me^]MgF [1.7977(11) Å] is considerably shorter than those with bridging fluoride ligands.^30^ For example, the Mg–F bond lengths of dinuclear {[BDI^Ar^]Mg(μ-F)(THF)}_2_ with μ_2_-bridges are 1.951(2) Å,^24^,^31^ while those of trinuclear [Mg_3_(μ_3_-F)(μ_2_-TFA)_6_(OMe)_2_(py)]^3–^ with a μ_3_-bridge range from 2.012(5) Å to 2.047(4) Å.^27^ Correspondingly, magnesium fluoride compounds that feature μ_4_-bridges exhibit even longer Mg–F bonds that range from 2.12 Å to 2.21 Å.^28^,^32^,^33^ In addition to the Mg–F bond of [Tp^Bu^t^,Me^]MgF being shorter than other Mg–F bonds, it is also amongst the shortest Mg–X (X ≠ H) bonds listed in the CSD, as illustrated by the magnesium oxide and alkoxide complexes, [{(THF)[BDI^Ar^]Mg}_2_(μ-O)] [1.8080(5) Å],^34^ [MesC{(C_4_N)Mes}_2_]Mg(OBu^t^)(THF) [1.804(2) Å],^35^ and [(ArO)Mg(μ-OAr)_2_]_2_Mg (Ar = C_6_H_3_Pr^i^_2_) [1.785(2) Å and 1.790(2) Å].^36^

Spectroscopically, [Tp^Bu^t^,Me^]MgF is characterized by a ^19^F NMR signal at –169.3 ppm, which is within the range exhibited by the related beryllium and zinc complexes, namely [Tp]BeF (–149 ppm),^37^ [Tp^Bu^t^,Me^]ZnF (–207 ppm),21*a* and [Tp^*p*-Tol,Me^]ZnF (–219 ppm),21*a* but is very different from the values observed for the dinuclear compounds, {[BDI^Ar^]Mg(μ-F) (THF)}_2_ (–25 ppm) and {[BDI^Ar^]Mg(μ-F)}_2_ (–26 ppm).^24^ While this large difference could be taken as an indication that ^19^F NMR spectroscopy could be used as a probe of fluoride coordination mode,^38^ we note that the chemical shift for [Tp^Bu^t^,Me^]MgF (–169.3 ppm) is also comparable to the solid state value for Mg_6_F_2_(OMe)_10_(MeOH)_14_ (–174.5 ppm), which contains μ_4_-F atoms.28*a* As such, it is evident that ^19^F NMR chemical shift data do not provide a definitive probe for the fluoride coordination mode in these systems. Nevertheless, ^19^F NMR data in a comparable region to that of [Tp^Bu^t^,Me^]MgF have been reported in protein systems;11c–h for example, PGM-MgF_3_-G6P-TSA in 100% H_2_O buffer exhibits ^19^F NMR chemical shifts of –147.0, –151.8, and –159.0 ppm.11*d*

The molecular structures of [Tp^Bu^t^,Me^]MgX (X = Cl, Br, I) have also been determined by X-ray diffraction.^39^ In each case, the molecules possess approximately *C*_3v_ symmetry, with a magnesium coordination geometry that is distorted considerably from tetrahedral. Specifically, the *τ*_4_ four-coordinate geometry indices^40^ range from 0.79 to 0.82 (Table 1) and deviate considerably from the value of 1.00 for that of an idealized tetrahedron.

**Table 1:** Metrical data for [Tp^Bu^t^,Me^]MgX

	*d*(M–X)/Å	*τ*_4_	B···M–X/°
[Tp^Bu^t^,Me^]MgF	1.7977(11)	0.79	177.8
[Tp^Bu^t^,Me^]MgCl	2.2701(15)	0.81	179.2
	2.2677(15)	0.81	179.1
[Tp^Bu^t^,Me^]MgBr	2.425(2)	0.81	178.9
	2.425(2)	0.82	179.0
[Tp^Bu^t^,Me^]MgI	2.6696(9)	0.80	177.8

**Fig. 2:**
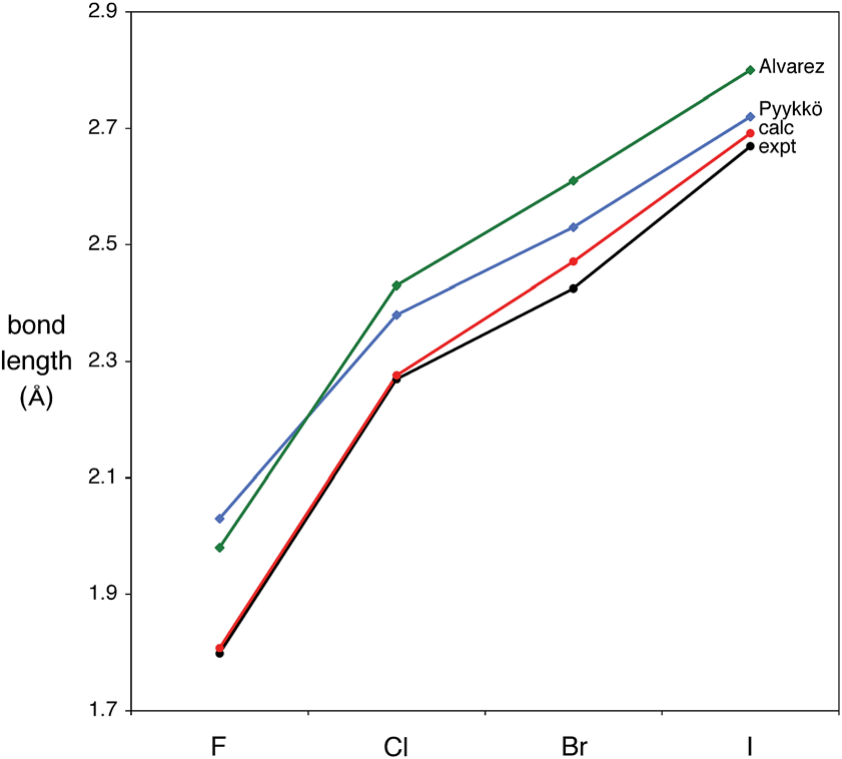
Comparison of experimental and calculated Mg–X (X = F, Cl, Br, I) bond lengths, together with the sum of Pyykkö and Alvarez covalent radii.

The availability of a complete series of structurally characterized halide compounds provides an opportunity to evaluate the bonding as a function of the halogen. The variation of the Mg–X bond lengths is illustrated in Table 1 and Fig. 2, which include, for comparison, the values predicted on the basis of the single bond covalent radii of the elements.

In this regard, it is pertinent to note that two sets of covalent radii have been recently proposed by Alvarez^41^ and Pyykkö,^42^,^43^ and that in each case the experimental Mg–X bond lengths are consistently smaller than those predicted by the sum of the covalent radii. With the exception of the fluoride derivative, the Pyykkö estimates are closer to the experimental bond lengths than are the Alvarez values. Specifically, the experimental Mg–X bond lengths are 0.13–0.19 Å shorter than the Alvarez values, and 0.05–0.23 Å shorter than the Pyykkö values. The magnesium–methyl bond length of [Tp^Bu^t^,Me^]MgMe [2.119(3) Å]^22^ is also shorter than the predicted values, although the difference (0.05 Å, Alvarez; 0.02 Å, Pyykkö) is much smaller than those for the halide derivatives. In addition to being smaller than the sum of the covalent radii, the experimental bond lengths are also shorter than the sum of the respective ionic radii.^44^

In principle, M–X bond lengths that are shorter than the sum of single-bond covalent radii can be a consequence of either (i) an ionic contribution to the bonding or (ii) π-bonding.^45^ To investigate this issue, we have examined the series of compounds, [Tp^Bu^t^,Me^]MgX (X = F, Cl, Br, I), computationally. Firstly, density functional theory (DFT) geometry optimization calculations reproduce the experimental structures very well, as indicated by the close correspondence between the experimental and calculated Mg–X bond lengths (Fig. 2). Secondly, the calculations indicate that the bonds have a significant ionic component, as illustrated by the atomic charges on the halogen, be they derived from Mulliken, electrostatic potential, or Natural population analysis. Thirdly, the bonds have no M–X π-interactions,^46^ such that it is the ionic component which provides a mechanism to shorten the Mg–X bond from that predicted by the sum of the covalent radii.^47^ Thus, both the experimental observations and the theoretical calculations are consistent with the Mg–X bonds having a significant ionic component; furthermore, the calculations indicate that this is greatest for the fluoride derivative (Table 2).

**Table 2:** Atomic charges (atomic units) on Mg and X in [Tp^Bu^t^,Me^]MgX (X = F, Cl, Br, I)

	NPA		Mulliken		ESP	
	*q*_Mg_/*e*	*q*_X_/*e*	*q*_Mg_/*e*	*q*_X_/*e*	*q*_Mg_/*e*	*q*_X_/*e*
F	1.733	–0.828	0.658	–0.496	0.334	–0.516
Cl	1.660	–0.809	0.526	–0.408	0.358	–0.431
Br	1.624	–0.767	0.516	–0.385	0.400	–0.426
I	1.597	–0.736	0.534	–0.400	0.448	–0.393

**Scheme 2:**
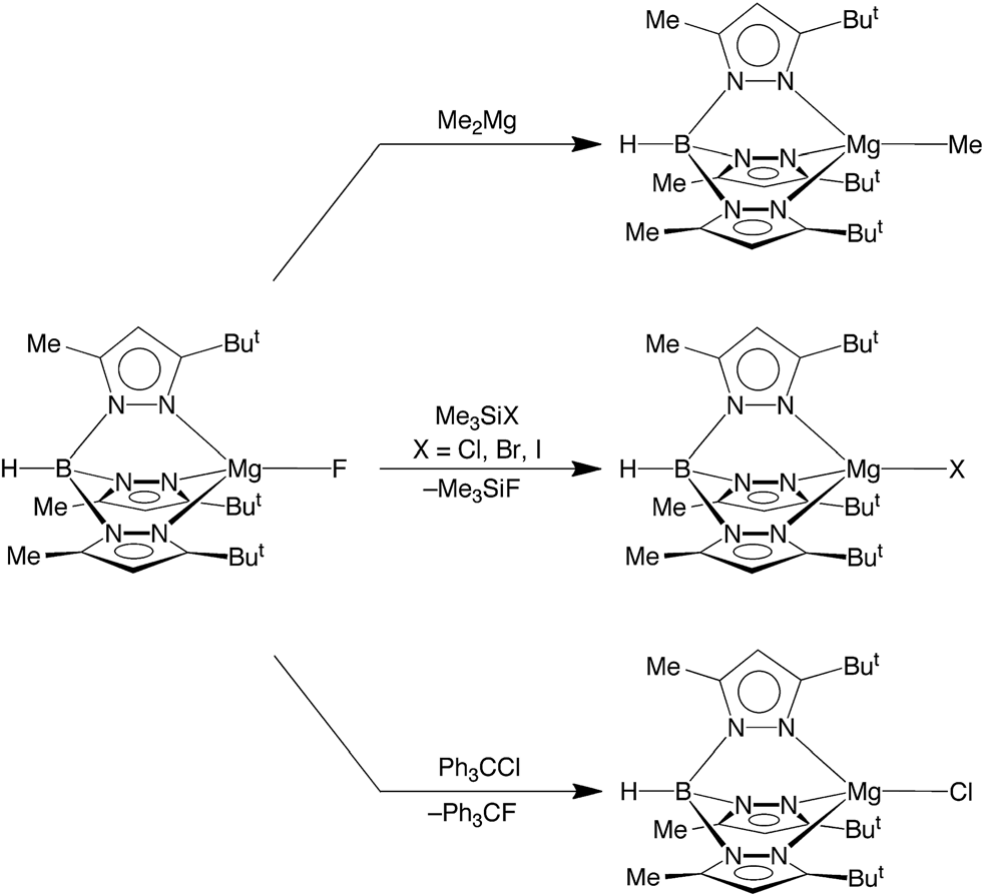
Reactivity of [Tp^Bu^t^,Me^]MgF.

In terms of reactivity, the fluoride compound [Tp^Bu^t^,Me^]MgF reacts with Me_2_Mg to regenerate the methyl derivative, [Tp^Bu^t^,Me^]MgMe (Scheme 2). Furthermore, the well known silaphilicity of fluorine^48^,^49^ provides a means to convert the fluoride complex [Tp^Bu^t^,Me^]MgF to the other halide derivatives *via* reaction with Me_3_SiX (X = Cl, Br, I),^50^ as illustrated in Scheme 2.

More interesting than its reactivity towards Me_3_SiX, [Tp^Bu^t^,Me^]MgF also reacts with Ph_3_CCl to afford [Tp^Bu^t^,Me^]MgCl and Ph_3_CF (Scheme 2). The ability of [Tp^Bu^t^,Me^]MgF to fluorinate Ph_3_CCl is of note because of the current significance of introducing fluorine into organic molecules,1a,^15^ which is of interest due to their role in pharmaceuticals and agrochemicals. The incorporation of fluorine into such molecules is, however, nontrivial, due to the facts that (i) fluoride has a large hydration energy and (ii) bonds to fluorine are strong.^1^ Therefore, considerable attention has been directed towards using metal-mediated transformations for introducing fluorine. The majority of studies, however, have focused on the use of transition metals.^15^ For example, [RuF(dppp)_2_]^+^ has also been used to convert Ph_3_CCl to Ph_3_CF.^51^ Thus, the corresponding reaction of [Tp^Bu^t^,Me^]MgF provides a novel example of C–F bond formation mediated by a covalent main group metal compound.

**Scheme 3:**
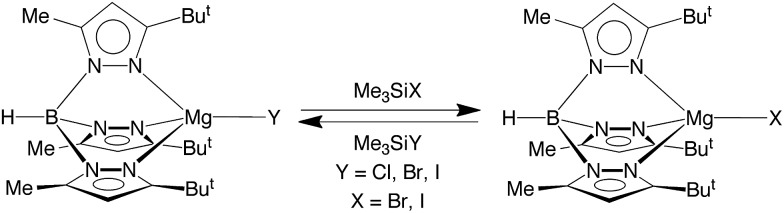
Halide exchange reactions.

In addition to [Tp^Bu^t^,Me^]MgF undergoing halogen exchange with Me_3_SiX (X = Cl, Br, I), the chloride and bromide complexes, [Tp^Bu^t^,Me^]MgCl and [Tp^Bu^t^,Me^]MgBr, also undergo halogen exchange with the heavier Me_3_SiX derivatives (Scheme 3). The magnitude of the equilibrium constants are such that they may be determined by NMR spectroscopy (Table 3), thereby indicating that the thermodynamics for the exchange between congeneric pairs of halogens, *i.e.* [Tp^Bu^t^,Me^]MgY (Y = F, Cl, Br) and Me_3_SiX (X = Cl, Br, I), becomes less exoergic upon descending the periodic table. The derived equilibrium constants for the reactions of [Tp^Bu^t^,Me^]MgY (Y = F, Cl, Br, I) with Me_3_SiI are also listed in Table 3, which indicates that the reaction which involves formation of the Si–F bond is more exoergic than that which involves formation of the Si–I bond. As such, the data provide quantitative evidence that the phenomenological silaphilicity of the halogens increases in the sequence I ≈ Br < Cl ≪ F. While this trend is in accord with the Si–F bond being stronger than the Si–I bond,^52^ it is important to emphasize that the thermodynamics are actually dictated by the relative values of Mg–X and Si–X bond energies.

**Table 3:** Thermodynamics for [Tp^Bu^t^,Me^]MgY/Me_3_SiX halogen exchange reactions

Reactants^*a*^	Products^*a*^	*K*
[Mg]F + Me_3_SiCl	[Mg]Cl + Me_3_SiF	>1000^*b*^
[Mg]Cl + Me_3_SiBr	[Mg]Br + Me_3_SiCl	13.4 ± 1.2^*b*^
[Mg]Br + Me_3_SiI	[Mg]I + Me_3_SiBr	0.93 ± 0.15^*b*^
[Mg]F + Me_3_SiI	[Mg]I + Me_3_SiF	>12 500^*c*^
[Mg]Cl + Me_3_SiI	[Mg]I + Me_3_SiCl	12.5^*c*^
[Mg]Br + Me_3_SiI	[Mg]I + Me_3_SiBr	0.93^*b*^
[Mg]I + Me_3_SiI	[Mg]I + Me_3_SiI	1^*d*^

^*a*^[Mg] = [Tp^Bu^t^,Me^]Mg.

^*b*^Experimental value.

^*c*^Derived from experimentally measured *K* values.

^*d*^Defined value.

Another interesting aspect of the reactivity of [Tp^Bu^t^,Me^]MgF pertains to its ability to participate in intermolecular interactions. In this regard, while fluorine is well recognized as an important structure-directing element by virtue of its ability to bridge two or more metal centers (*vide supra*),^53^ it may also serve a structural role by participating in hydrogen bonding^54^ and halogen bonding^55^–^57^ interactions. The latter is a directional attractive noncovalent interaction between a covalently bound halogen atom (X), *e.g.* R–X or X–X, and a Lewis base, and results from the electron density distribution about X being anisotropic, such that it creates a belt of high electron density perpendicular to the covalent bond, but a region of low electron density (a so-called σ-hole) in the direction of the bond.^55^ Albeit much less heavily investigated than hydrogen bonding, halogen bonding has been shown to be an important tool in crystal engineering,^55^ with geometrical preferences that are similar to hydrogen bonding interactions, *i.e.* linear A···X–D motifs, where A is the acceptor for the halogen bond and D is the donor. However, despite many structural investigations pertaining to intermolecular interactions involving metal fluoride ligands,54b,g there are few reports that detail the thermodynamics associated with either hydrogen bonding,50b,^58^–^60^ or halogen bonding interactions.50b,^58^,^61^,^62^ Therefore, we have examined the ability of the fluoride ligand of [Tp^Bu^t^,Me^]MgF to serve as a hydrogen bond and halogen bond acceptor.

**Scheme 4:**
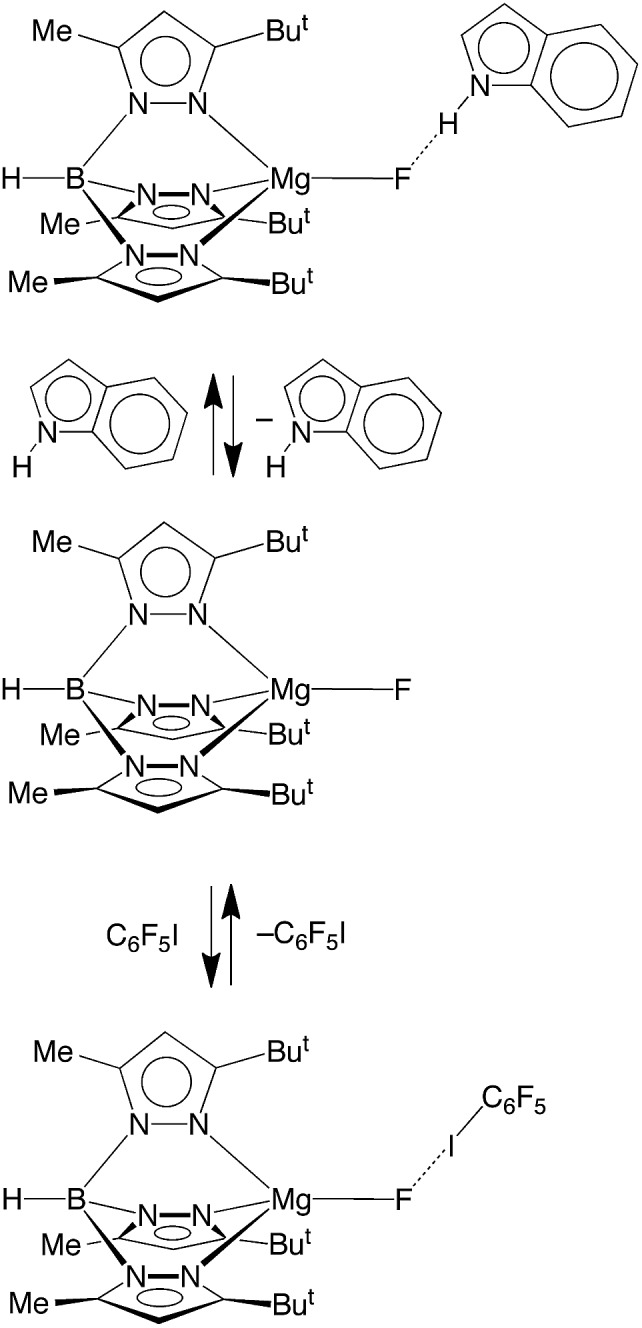
Hydrogen and halogen bonding interactions of [Tp^Bu^t^,Me^]MgF.

**Fig. 3:**
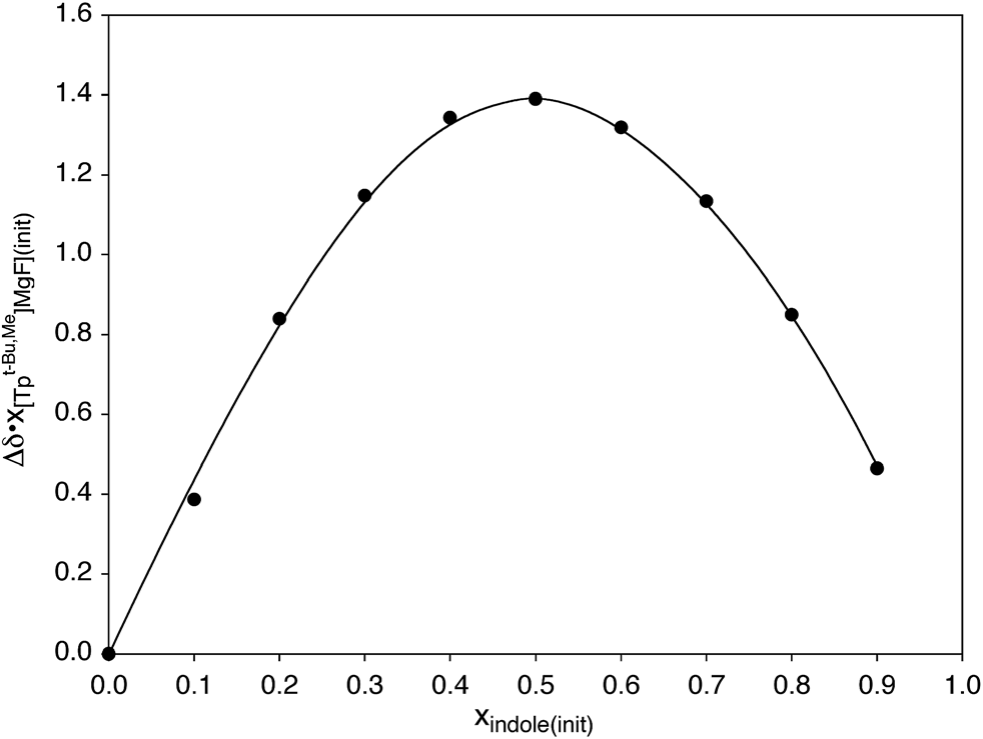
Job plot for coordination of indole to [Tp^Bu^t^,Me^]MgF as measured by ^1^H NMR spectroscopy.

Hydrogen bonding interactions involving magnesium fluoride species are of relevance to the use of *in situ* generated [MgF_3_]^–^ to provide transition state analogues of phosphoryl transfer.^11^,^12^,^63^ In this regard, indole is a useful probe for quantitative studies because, although it is a good hydrogen bond donor, it is neither a good hydrogen bond acceptor nor a good nitrogen donor ligand,^58^,^64^ both of which would otherwise complicate the analysis. In this regard, Job plots^65^ based on ^1^H and ^19^F NMR spectroscopic data demonstrate that the interaction between [Tp^Bu^t^,Me^]MgF and indole involves formation of a 1 : 1 adduct in benzene (Scheme 4 and Fig. 3).^66^ Analysis of the variation of the ^19^F NMR chemical shift as a function of indole concentration provides a binding constant of *K* = 39 ± 6 M^–1^ at 300 K for formation of the 1 : 1 adduct, [Tp^Bu^t^,Me^]MgF·indole.^67^ For comparison, there are few reports pertaining to the thermodynamics of hydrogen bonding of indole to a terminal fluoride ligand, namely [κ^4^-Tptm]ZnF (85 M^–1^),50*b* (Et_3_P)_2_Ni(C_5_NF_4_)F (57.9 M^–1^),^58^,^68^ and Cp*_2_MF_2_ (M = Ti, 5.4 M^–1^; M = Zr, 1.4 M^–1^; M = Hf, 1.4 M^–1^),^69^ from which it is evident that [Tp^Bu^t^,Me^]MgF must be considered a significant hydrogen bond acceptor.

The ability of [Tp^Bu^t^,Me^]MgF to participate in halogen bonding interactions has been investigated by a related study using C_6_F_5_I. Thus, ^19^F NMR spectroscopy demonstrates that the fluoride ligand of [Tp^Bu^t^,Me^]MgF serves as a halogen bond acceptor (Scheme 4) with the ^19^F NMR chemical signal shifting downfield upon addition of C_6_F_5_I.^70^ The derived binding constant (1.6 ± 0.3 M^–1^) is approximately an order of magnitude smaller than the hydrogen bonding interaction involving indole, but is comparable to the few reports of halogen bonding interactions involving fluoride ligands, namely [κ^4^-Tptm]ZnF (9.0 M^–1^) and *trans*-(R_3_P)_2_M(Ar)F (M = Ni, Pd, Pt; 2.4 to 5.2 M^–1^).^58^,61a

## Conclusions

In summary, the first structurally characterized example of a molecular magnesium compound that features a terminal fluoride ligand, namely [Tp^Bu^t^,Me^]MgF, has been obtained by the reaction of [Tp^Bu^t^,Me^]MgMe with Me_3_SnF. The chloride, bromide and iodide complexes, [Tp^Bu^t^,Me^]MgX, can also be obtained by analogous methods using Me_3_SnX (X = Cl, Br, I). Structural characterization by X-ray diffraction demonstrates that, in each case, the Mg–X bond lengths are shorter than the sum of the covalent radii, thereby indicating that there is a significant ionic component to the bonding, which is in accord with density functional theory calculations.

The fluoride ligand of [Tp^Bu^t^,Me^]MgF undergoes halide exchange with Me_3_SiX (X = Cl, Br, I) to afford [Tp^Bu^t^,Me^]MgX. The other halide derivatives [Tp^Bu^t^,Me^]MgX undergo similar exchange reactions, but the thermodynamic driving forces are much smaller than those involving fluoride transfer, a manifestation of the often discussed silaphilicity of fluorine. [Tp^Bu^t^,Me^]MgF also undergoes metathesis with Ph_3_CCl to afford Ph_3_CF, thereby demonstrating that [Tp^Bu^t^,Me^]MgF has applications in the formation of C–F bonds.

In accord with the highly polarized nature of the Mg–F bond, the fluoride ligand of [Tp^Bu^t^,Me^]MgF is capable of serving as a hydrogen bond and halogen bond acceptor to indole and C_6_F_5_I, respectively. The ability of [Tp^Bu^t^,Me^]MgF to participate in hydrogen bonding interactions mimics the involvement of magnesium fluoride species in biological systems.

## Supplementary Material

Supplementary information

Crystal structure data
