# Supplementary material for: Synthesis, structure and reactivity of a terminal magnesium fluoride compound, [TpBut,Me]MgF: hydrogen bonding, halogen bonding and C–F bond formation
Source: Chem Sci. 2015 Nov 17;7(1):142–9. doi: 10.1039/c5sc03504j (PMC5950829; doi:10.1039/c5sc03504j)
Supplement: Supplementary file 1 [file SC-007-C5SC03504J-s001.pdf]

Electronic Supplementary Material (ESI) for Chemical Science.
This journal is © The Royal Society of Chemistry 2015

SUPPORTING INFORMATION

Synthesis, Structure and Reactivity of a Terminal Magnesium Fluoride Compound, [$Tp^{Bu^t,Me}$]MgF: Hydrogen Bonding, Halogen Bonding and C–F Bond Formation

Michael Rauch, Serge Ruccolo, John Paul Mester, Yi Rong, Gerard Parkin,*

Department of Chemistry,

Columbia University,

New York, New York 10027, USA.

Received xxxx xx, 2015.

EXPERIMENTAL SECTION

General considerations

All manipulations were performed using a combination of glovebox, high vacuum, and Schlenk techniques under an argon atmosphere.[1] Solvents were purified and degassed using standard procedures. NMR spectra were measured on Bruker 300 DRX, Bruker 300 DPX, Bruker 400 Avance III, Bruker 400 Cyber-enabled Avance III, and Bruker 500 DMX spectrometers. ^{1}H NMR spectra are reported in ppm relative to $SiMe_4$ ($\delta = 0$) and were referenced internally with respect to the protio solvent impurity ($\delta = 7.16$ for C_6D_5H and 7.09 for C_7D_7H).[2] ^{13}C NMR spectra are reported in ppm relative to $SiMe_4$ ($\delta = 0$) and were referenced internally with respect to the solvent ($\delta = 128.06$ for C_6D_6).[2] ^{11}B NMR are reported in ppm relative to $BF_3{\bullet}Et_2O$ ($\delta = 0$) and were obtained by using the $\Xi/100\%$ value of 32.083974.[3] ^{19}F NMR chemical shifts are reported in ppm relative to $CFCl_3$ ($\delta = 0.0$) and were obtained by using the $\Xi/100\%$ value of 94.094011.[3] Infrared spectra were recorded on a Perkin Elmer Spectrum Two spectrometer in attenuated total reflectance (ATR) mode, and are reported in reciprocal centimeters. $[Tp^{Bu^t,Me}]Li$,[4] Me_2Mg,[5] Me_3SnF,[6] and Me_3SnI,[7] were obtained by literature methods and Me_3SnCl (Strem Chemicals), Me_3SnBr (Alfa Aesar), Me_3SiCl (Sigma Aldrich), Me_3SiBr (TCI), Me_3SiI (Alfa Aesar), Ph_3CCl (Sigma Aldrich), indole (Sigma Aldrich) and C_6F_5I (Strem Chemicals) were obtained commercially and used as received.

X-ray Structure Determinations

X-ray diffraction data were collected on a Bruker Apex II diffractometer. Crystal data, data collection and refinement parameters are summarized in Table 1. The structure was solved by using direct methods and standard difference map techniques, and was refined by full-matrix least-squares procedures on F^2 with SHELXTL (Version 2014/7).[8]

Computational Details

Calculations were carried out using DFT as implemented in the Jaguar 7.7 (release 107) suite of *ab initio* quantum chemistry programs.[9] Geometry optimizations were performed with the B3LYP density functional[10] using the LACVP** basis sets. The energies of the optimized structures were re-evaluated by additional single point calculations on each optimized geometry using the cc-pVTZ(-f) correlation consistent triple (all atoms except I) and LACV3P** (I) basis sets.[11] Cartesian coordinates for geometry optimized structures are listed in Table 2.

Synthesis of [Tp$^{Bu^t,Me}$]MgMe

A solution of [Tp$^{Bu^t,Me}$]Li (500 mg, 1.162 mmol) in benzene (7 mL) was treated with Me_2Mg (250 mg, 4.598 mmol) and stirred for twelve hours at 80 °C. After this period, the mixture was filtered and the solution was lyophilized to afford [Tp$^{Bu^t,Me}$]MgMe as a white powder (384 mg, 71% yield), which was identified by comparison of the ^{1}H NMR spectroscopic data with that of the literature[12] and by single crystal X-ray diffraction on crystals obtained from a benzene solution. ^{11}B{^{1}H} NMR (C_6D_6): -8.8 [br, $\underline{B}$H].

Synthesis of [Tp$^{Bu^t,Me}$]MgF

A solution of [Tp$^{Bu^t,Me}$]MgMe (100 mg, 0.216 mmol) in benzene (3 mL) was treated with Me_3SnF (45 mg, 0.246 mmol) and the mixture was stirred for 2.5 hours at room temperature . After this period, the mixture was filtered and the solution was lyophilized to afford [Tp$^{Bu^t,Me}$]MgF as a white powder (90 mg, 89% yield). Colorless crystals of [Tp$^{Bu^t,Me}$]MgF suitable for X-ray diffraction were obtained *via* slow evaporation from a benzene solution. Anal. calcd. for [Tp$^{Bu^t,Me}$]MgF: C, 61.8%; H, 8.6% N, 18.0%. Found: C, 62.1%; H, 8.8%; N, 18.1%. ^{1}H NMR (C_6D_6): 1.52 [s, 27H of 3(C(C$\underline{H}_3$)$_3$)], 2.11 [s, 9H of 3(C$\underline{H}_3$)], 4.83 [br, 1H of $\underline{H}$B], 5.63 [s, 3H of 3($C_3N_2\underline{H}$)]. ^{13}C{^{1}H} NMR (C_6D_6): 12.70 [s, 3C of 3($\underline{C}H_3$)], 30.87 [s, 9C of 3(C($\underline{C}H_3$)$_3$)], 31.99 [s, 3C of 3($\underline{C}$(CH$_3$)$_3$)], 103.03 [s, 3C of 3($\underline{C}_3N_2H$)], 144.88 [s, 3C of 3($\underline{C}_3N_2H$)], 164.49 [s, 3C of

3($\underline{C}_3N_2H$)]. $^{11}B\{^1H\}$ NMR (C_6D_6): -9.3 [br, $\underline{B}$H]. $^{19}F\{^1H\}$ NMR (C_6D_6): -169.3 [s, Mg$\underline{F}$]. ^1H NMR (C_7D_8): 1.46 [s, 27H of 3(C(C$\underline{H}_3$)$_3$)], 2.11 [s, 9H of 3(C$\underline{H}_3$)], 4.70 [br, 1H of $\underline{H}$B], 5.61 [s, 3H of 3($C_3N_2\underline{H}$)]. $^{19}F\{^1H\}$ NMR (C_7D_8): -173.1 [s, Mg$\underline{F}$]. IR Data (ATR, cm^{-1}): 2956 (m), 1540 (m), 1466 (w), 1429 (m), 1384 (w), 1364 (m), 1346 (w), 1243 (w), 1184 (vs), 1066 (vs), 1031 (m), 986 (w), 854 (w), 801 (w), 786 (s), 763 (vs), 680 (m), 648 (vs), 520 (m), 464 (w), 446 (w), 428 (w). Although density functional theory calculations predict a value of 708 cm^{-1} for ν(Mg–F), it is not possible to make a definitive assignment in the experimental spectrum.

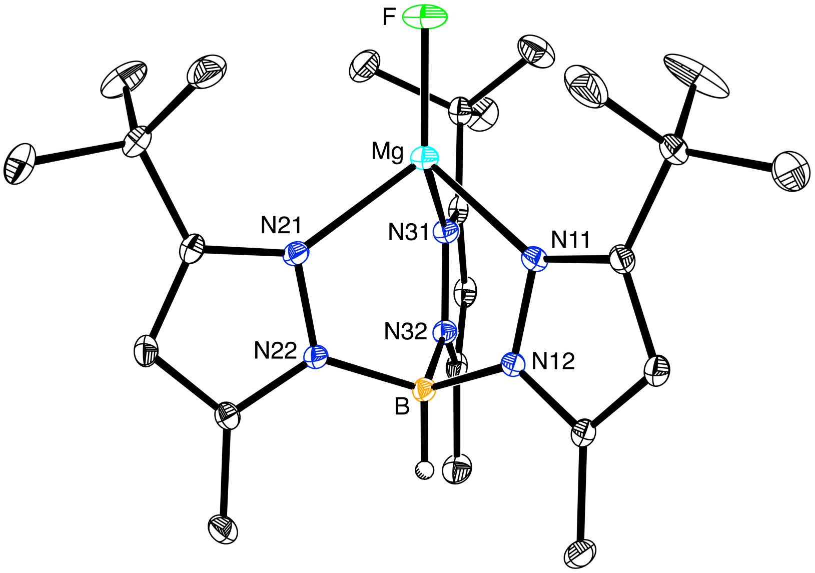

Molecular Structure of [Tp$^{Bu^t,Me}$]MgF

Synthesis of [Tp$^{Bu^t,Me}$]MgCl

A solution of [Tp$^{Bu^t,Me}$]MgMe (10 mg, 0.022 mmol) in benzene (0.7 mL) was treated with Me_3SnCl (5 mg, 0.025 mmol), resulting in the formation of a white precipitate over a period of 10 minutes. After this period, the solution was lyophilized and the product washed with pentane to afford [Tp$^{Bu^t,Me}$]MgCl as a white powder (7 mg, 67% yield). Colorless crystals suitable for X-ray diffraction were obtained *via* slow evaporation from a benzene solution. Anal. calcd. for [Tp$^{Bu^t,Me}$]MgCl•0.5C_6H_6: C, 62.1%; H, 8.3%; N, 16.1%. Found: C, 62.2%; H, 8.3%; N, 16.3%. ^1H NMR (C_6D_6): 1.55 [s, 27H of 3(C(C$\underline{H}_3$)$_3$)], 2.09 [s,

9H of $3(C\underline{H}_3)$], 4.72 [br, 1H of $\underline{H}B$], 5.64 [s, 3H of $3(C_3N_2\underline{H})$]. $^{13}C\{^1H\}$ NMR (C_6D_6): 12.81 [s, 3C of $3(\underline{C}H_3)$], 31.19 [s, 9C of $3(C(\underline{C}H_3)_3)$], 32.08 [s, 3C of $3(\underline{C}(CH_3)_3)$], 103.60 [s, 3C of $3(\underline{C}_3N_2H)$], 145.03 [s, 3C of $3(\underline{C}_3N_2H)$], 165.18 [s, 3C of $3(\underline{C}_3N_2H)$]. ^{11}B NMR (C_6D_6): -8.9 [br, $\underline{B}H$]. IR Data (ATR, cm^{-1}): 2956 (m), 1541 (s), 1474 (m), 1428 (m), 1385 (w), 1363 (m), 1341 (m), 1242 (m), 1186 (vs), 1176 (s), 1068 (s), 1031 (m), 988 (w), 853 (w), 802 (m), 787 (s), 778 (vs), 736 (w), 681 (w), 650 (vs), 520 (m), 508 (s) 464 (s), 427 (s).

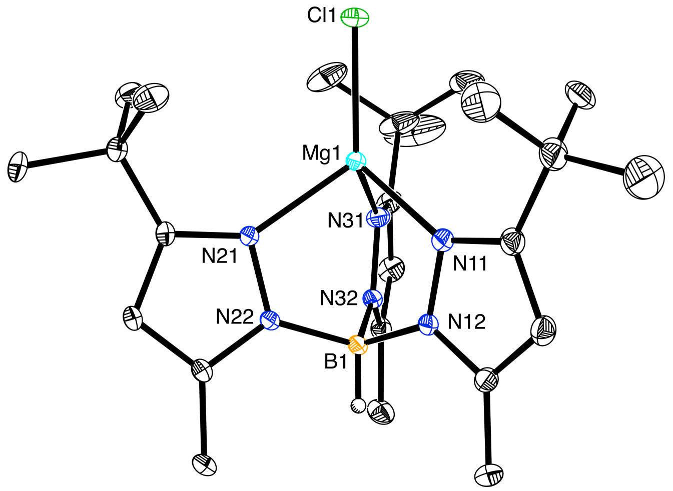

Molecular Structure of [Tp$^{Bu^t,Me}$]MgCl

Synthesis of [Tp$^{Bu^t,Me}$]MgBr

A solution of [Tp$^{Bu^t,Me}$]MgMe (10 mg, 0.022 mmol) in benzene (0.7 mL) was treated with Me_3SnBr (7 mg, 0.029 mmol), resulting in the formation of a white precipitate over a period of 10 minutes. After this period, the solution was lyophilized and the product washed with pentane to afford [Tp$^{Bu^t,Me}$]MgBr as a white powder (8 mg, 70% yield). Colorless crystals suitable for X-ray diffraction were obtained *via* slow evaporation from a benzene solution. Anal. calcd. for [Tp$^{Bu^t,Me}$]MgBr•$0.5C_6H_6$: C, 57.1%; H, 7.7%; N, 14.8%. Found: C, 56.4%; H, 7.6%; N, 14.5%. ^1H NMR (C_6D_6): 1.57 [s, 27H of $3(C(C\underline{H}_3)_3)$], 2.08 [s, 9H of $3(C\underline{H}_3)$], 4.78 [br, 1H of $\underline{H}B$], 5.64 [s, 3H of $3(C_3N_2\underline{H})$]. $^{13}C\{^1H\}$ NMR (C_6D_6): 12.86 [s, 3C of $3(\underline{C}H_3)$], 31.45 [s, 9C of $3(C(\underline{C}H_3)_3)$], 32.15 [s, 3C of $3(\underline{C}(CH_3)_3)$], 103.76 [s, 3C of $3(\underline{C}_3N_2H)$], 145.10 [s, 3C of $3(\underline{C}_3N_2H)$], 165.38 [s, 3C of $3(\underline{C}_3N_2H)$]. $^{11}B\{^1H\}$ NMR (C_6D_6): -

8.9 [br, B̲H]. IR Data (ATR, cm^{-1}): 2957 (m), 2570 (w), 1541 (s), 1474 (m), 1425 (s), 1385 (w), 1363 (s), 1338 (w), 1241 (w), 1187 (vs), 1176 (s), 1068 (vs), 1030 (m), 987 (w), 800 (m), 788 (s), 778 (s), 766 (vs), 680 (w), 650 (vs), 520 (m), 506 (s) 453 (s), 425 (w).

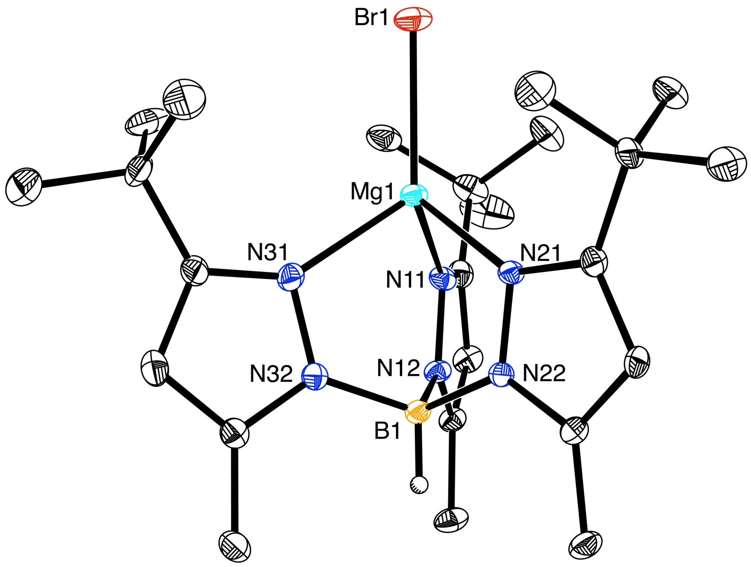

Molecular Structure of [Tp$^{Bu^t,Me}$]MgBr

Synthesis of [Tp$^{Bu^t,Me}$]MgI

A solution of [Tp$^{Bu^t,Me}$]MgMe (10 mg, 0.022 mmol) in benzene (0.7 mL) was treated with Me_3SnI (*ca.* 5 equiv.), resulting in the formation of a white precipitate over a period of 10 minutes. After this period, the mixture was lyophilized and the product washed with pentane to afford [Tp$^{Bu^t,Me}$]MgI as a white powder (8 mg, 64% yield). Colorless crystals suitable for X-ray diffraction were obtained *via* slow evaporation from a benzene solution. Anal. calcd. for [Tp$^{Bu^t,Me}$]MgI: C, 50.1%; H, 7.0%; N, 14.6%. Found: C, 50.0%; H, 7.1%; N, 14.5%. ^{1}H NMR (C_6D_6): 1.59 [s, 27H of 3(C(C$\underline{H}_3$)$_3$)], 2.07 [s, 9H of 3(C$\underline{H}_3$)], 4.78 [br, 1H of H̲B], 5.63 [s, 3H of 3($C_3N_2\underline{H}$)]. ^{13}C{^{1}H} NMR (C_6D_6): 12.93 [s, 3C of 3($\underline{C}H_3$)], 32.02 [s, 9C of 3(C($\underline{C}H_3$)$_3$)], 32.30 [s, 3C of 3($\underline{C}$(CH$_3$)$_3$)], 103.97 [s, 3C of 3($\underline{C}_3N_2H$)], 145.19 [s, 3C of 3($\underline{C}_3N_2H$)], 165.62 [s, 3C of 3($\underline{C}_3N_2H$)]. ^{11}B{^{1}H} NMR (C_6D_6): -9.2 [br, B̲H]. IR Data (ATR, cm^{-1}): 2954 (w), 2560 (w), 1542 (m), 1532 (m), 1473 (w), 1422 (m), 1382 (w),

1358 (m), 1334 (w), 1238 (w), 1186 (vs), 1174 (s), 1130 (w), 1065 (vs), 1021 (m), 986 (w), 862 (w), 850 (s), 841 (w), 764 (vs), 678 (w), 646 (s), 519 (w), 449 (w).

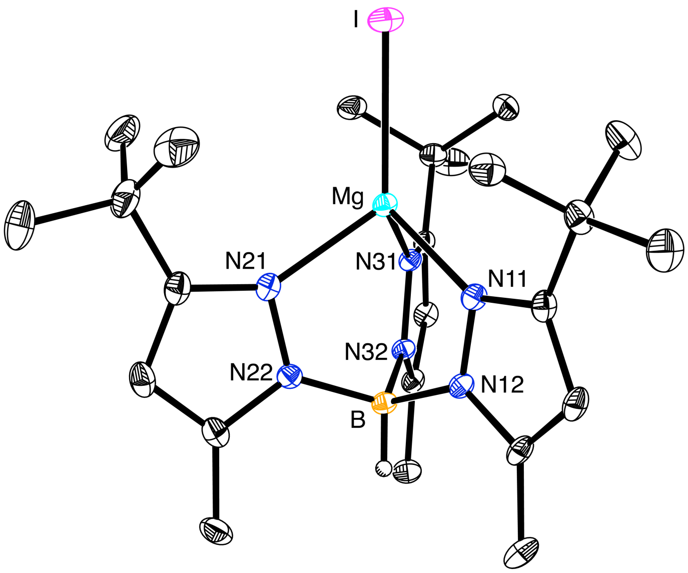

Molecular Structure of [Tp$^{Bu^t,Me}$]MgI

Reactivity of [Tp$^{Bu^t,Me}$]MgF towards Me_2Mg

A solution of [Tp$^{Bu^t,Me}$]MgF (10 mg, 0.021 mmol) in benzene (0.7 mL) was treated with Me_2Mg (5 mg, 0.092 mmol). The mixture was filtered after one hour and the solution was lyophilized to afford [Tp$^{Bu^t,Me}$]MgMe as a white powder (8 mg, 81% yield) as identified by ^1H NMR spectroscopy.

Reactivity of [Tp$^{Bu^t,Me}$]MgF towards Me_3SiX (X = Cl, Br, I)

A solution of [Tp$^{Bu^t,Me}$]MgF (2 mg, 0.004 mmol) in C_6D_6 (0.7 mL) in an NMR tube equipped with a J. Young valve was treated with excess Me_3SiX (X = Cl, Br, I; *ca.* 3 equiv.) *via* vapor transfer and the sample was monitored by ^1H and ^{19}F NMR spectroscopy, thereby demonstrating the formation of [Tp$^{Bu^t,Me}$]MgX and Me_3SiF.

Reactivity of [Tp$^{Bu^t,Me}$]MgY (Y = Cl, Br, I) towards Me_3SiX (X = Cl, Br, I)

A solution [Tp$^{Bu^t,Me}$]MgY (2 mg) in C_6D_6 (0.7 mL) in an NMR tube equipped with a J. Young valve was treated with a solution of Me_3SiX in C_6D_6 (*ca.* 1 equiv). The sample was monitored by ^{1}H NMR spectroscopy, thereby demonstrating the formation of an equilibrium mixture containing [Tp$^{Bu^t,Me}$]MgX and Me_3SiY over a period of 2 hours. Integration of the [Tp$^{Bu^t,Me}$]MgY, [Tp$^{Bu^t,Me}$]MgX, Me_3SiX and Me_3SiY components allowed measurement of the equilibrium constants. Similar experiments were performed with different concentrations of reactants to obtain the average equilibrium constant.

Reactants	Products	K^a
[Tp$^{Bu^t,Me}$]MgF + Me_3SiCl	[Tp$^{Bu^t,Me}$]MgCl + Me_3SiF	> 1,000
[Tp$^{Bu^t,Me}$]MgCl + Me_3SiBr	[Tp$^{Bu^t,Me}$]MgBr + Me_3SiCl	13.4 ± 1.2
[Tp$^{Bu^t,Me}$]MgBr + Me_3SiI	[Tp$^{Bu^t,Me}$]MgI + Me_3SiBr	0.93 ± 0.15

Reactivity of [Tp$^{Bu^t,Me}$]MgF towards Ph_3CCl

A solution of [Tp$^{Bu^t,Me}$]MgF (3 mg, 0.006 mmol) in benzene (0.7 mL) in an NMR tube equipped with a J. Young valve was treated with Ph_3CCl (2 mg, 0.007 mmol). The mixture was heated at 80 ˚C for 48 hours to afford [Tp$^{Bu^t,Me}$]MgCl and Ph_3CF as identified by comparison of the ^{1}H and ^{19}F NMR spectra to those of authentic samples.[13]

Hydrogen Bonding of Indole to [Tp$^{Bu^t,Me}$]MgF

(a) Equimolar solutions of *(i)* [Tp$^{Bu^t,Me}$]MgF in C_6D_6 (30 mM) and *(ii)* indole in C_6D_6 (30 mM) were prepared by using mesitylene as an internal standard. The solutions were combined in different ratios, such that the total volume was kept constant in all the samples. The samples were monitored by ^{1}H and ^{19}F NMR spectroscopies and the stoichiometry of the [Tp$^{Bu^t,Me}$]MgF•••indole adduct was obtained from a Job plot of $\Delta\delta x_{F(init)}$ *versus* $x_{I(init)}$, where $\Delta\delta = \delta\{[Tp^{Bu^t,Me}]MgF\} - \delta_{obs}$, and $x_{F(init)}$ and $x_{I(init)}$ are

respectively the mole fractions of [Tp$^{Bu^t,Me}$]MgF and indole that are present prior to the establishment of equilibration. For analysis by ^{1}H NMR spectroscopy, Δδ refers to the change in chemical shift of the signal at δ 1.52 in pure [Tp$^{Bu^t,Me}$]MgF; for analysis by ^{19}F NMR spectroscopy, Δδ refers to the change in chemical shift of the signal at δ –169.3 in pure [Tp$^{Bu^t,Me}$]MgF. The observation that the maximum occurs at a mole fraction of *ca.* 0.5 for both the ^{1}H and ^{19}F NMR spectroscopic data indicates that the adduct has a 1:1 composition.

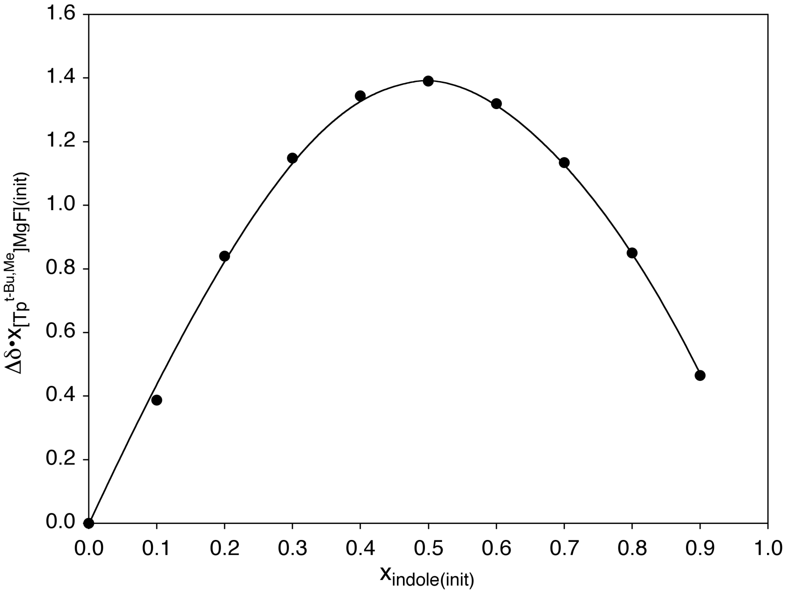

Job plot for coordination of indole to [Tp$^{Bu^t,Me}$]MgF as measured by ^{1}H NMR spectroscopy.

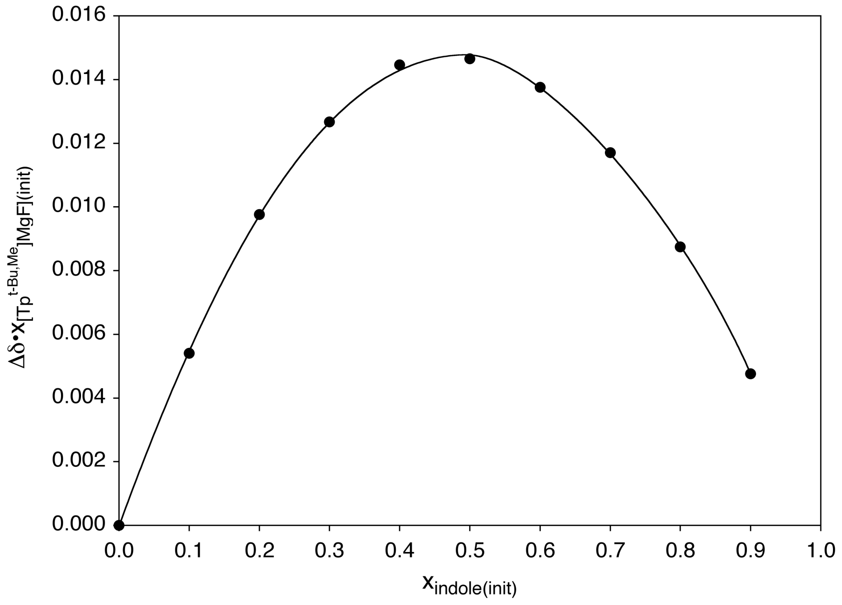

Job plot for coordination of indole to [Tp$^{Bu^t,Me}$]MgF as measured by ^{19}F NMR spectroscopy.

(b) A solution of [Tp$^{Bu^t,Me}$]MgF in C_6D_6 (0.4 mL of 30 mM) was treated with aliquots of a solution of indole in C_6D_6 (66 mM) and monitored by ^{19}F NMR spectroscopy, as illustrated below. The equilibrium constant for coordination of indole [35 ± 1 M^{-1}, 43 ± 2; average = 39 ± 6 M^{-1}] was determined by using WinEQNMR2[14] to fit the ^{19}F NMR chemical shift as a function of total [Tp$^{Bu^t,Me}$]MgF and total indole concentration, whether coordinated or uncoordinated.

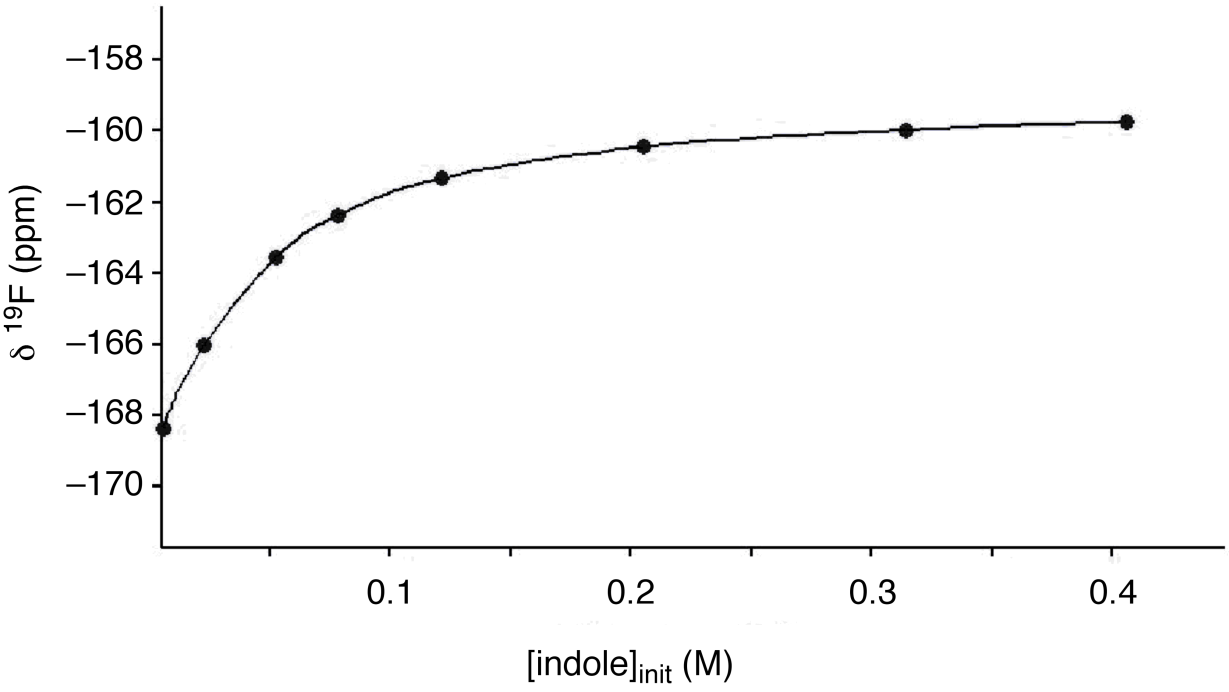

Variation of the ^{19}F NMR chemical shift of $[Tp^{Bu^t,Me}]MgF$ as a function of indole concentration (the line corresponds to the fit by WinEQNMR2; $K = 35 \pm 1\ M^{-1}$).

Halogen Bonding of C_6F_5I to $[Tp^{Bu^t,Me}]MgF$

A solution of $[Tp^{Bu^t,Me}]MgF$ in C_6D_6 (30 mM) was treated with aliquots of a solution of C_6F_5I in C_6D_6 (67 mM) and monitored by ^1H and ^{19}F NMR spectroscopies, as illustrated below. The equilibrium constant for coordination of C_6F_5I [1.4 ± 0.1, 1.7 ± 0.2; average = $1.5 \pm 0.4\ M^{-1}$] was determined by using WinEQNMR2[14] to fit ^{19}F NMR chemical shift as a function of total $[Tp^{Bu^t,Me}]MgF$ and total C_6F_5I concentration, whether coordinated or uncoordinated.

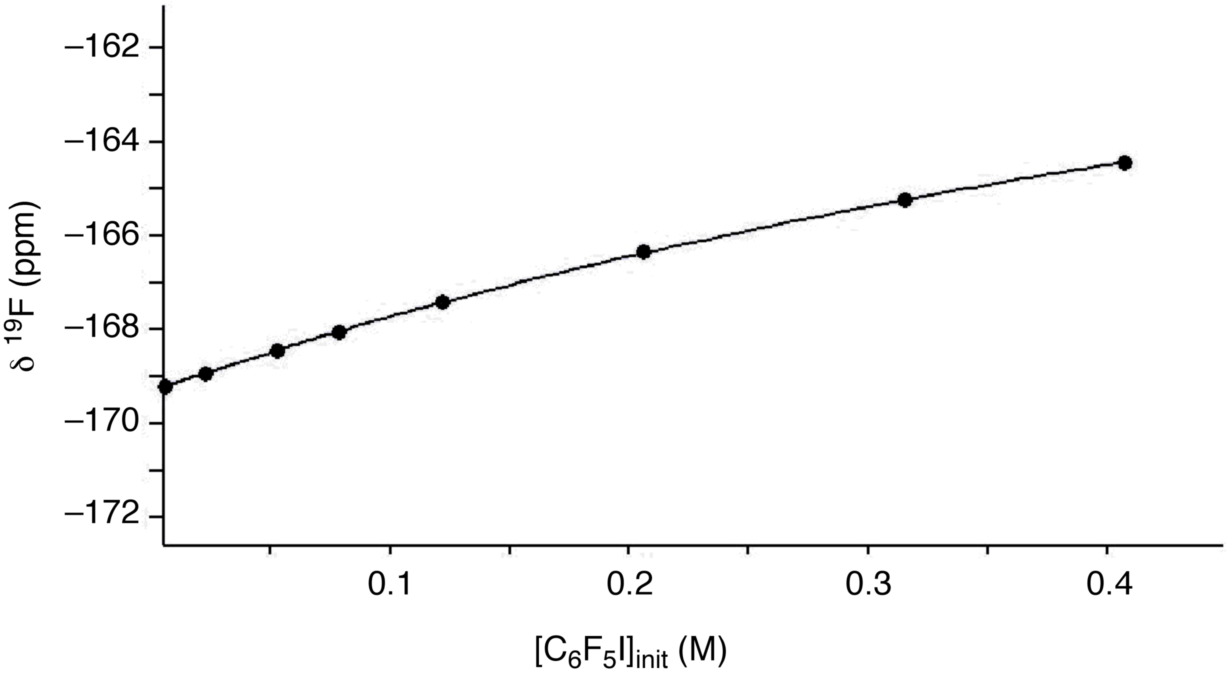

Variation of the ${}^{19}F$ NMR chemical shift of $[Tp^{Bu^t,Me}]MgF$ as a function of total C_6F_5I concentration (the line corresponds to the fit by WinEQNMR2; $K = 1.3 \pm 0.2\ M^{-1}$).

Table 1. Crystal, intensity collection, and refinement data.

	**[Tp$^{Bu^t,Me}$]MgF**	**[Tp$^{Bu^t,Me}$]MgCl•0.5C_6H_6**
lattice	Monoclinic	Monoclinic
formula	$C_{24}H_{40}BFN_6Mg$	$C_{27}H_{43}BClN_6Mg$
formula weight	466.74	522.24
space group	$P2_1/n$	$P2_1$
a/Å	9.422(3)	9.726(5)
b/Å	30.397(9)	17.928(9)
c/Å	9.544(3)	17.689(9)
α/°	90	90
β/°	98.992(4)	90.136(8)
γ/°	90	90
V/Å^3	2699.9(14)	3084(3)
Z	4	4
temperature (K)	130(2)	130(2)
radiation (λ, Å)	0.71073	0.71073
ρ (calcd.) g cm^{-3}	1.148	1.125
μ (Mo Kα), mm^{-1}	0.095	0.169
θ max, deg.	30.563	30.539
no. of data collected	43077	50136
no. of data	8255	18702
no. of parameters	327	706
R_1 [$I > 2\sigma(I)$]	0.0507	0.0541
wR_2 [$I > 2\sigma(I)$]	0.1234	0.1405
R_1 [all data]	0.0696	0.0667
wR_2 [all data]	0.1336	0.1531
GOF	1.036	1.048
R_{int}	0.0653	0.0626
Abs. struct. param.	–	–0.01(3)

Table 1 (cont). Crystal, intensity collection, and refinement data.

	**[Tp$^{Bu^t,Me}$]MgBr•0.5C$_6$H$_6$**	**[Tp$^{Bu^t,Me}$]MgI**
lattice	Monoclinic	Monoclinic
formula	$C_{27}H_{43}BBrN_6Mg$	$C_{24}H_{40}BIN_6Mg$
formula weight	566.70	574.64
space group	$P2_1$	$P2_1/n$
a / Å	9.724(4)	10.469(3)
b / Å	18.118(7)	16.987(5)
c / Å	17.751(6)	16.162(5)
α / °	90	90
β / °	90.132(6)	91.359(4)
γ / °	90	90
V / Å^3	3127(2)	2873.5(14)
Z	4	4
temperature (K)	130(2)	130(2)
radiation (λ, Å)	0.71073	0.71073
ρ (calcd.) g cm^{-3}	1.204	1.328
μ (Mo Kα), mm^{-1}	1.359	1.158
θ max, deg.	30.679	30.659
no. of data collected	50225	46949
no. of data	19088	8857
no. of parameters	644	310
R_1 [$I > 2\sigma(I)$]	0.0653	0.0387
wR_2 [$I > 2\sigma(I)$]	0.1710	0.0927
R_1 [all data]	0.0880	0.0550
wR_2 [all data]	0.1845	0.1012
GOF	1.076	1.032
R_{int}	0.0714	0.0512
Abs. struct. param.	0.009(5)	–

Table 2. Cartesian Coordinates for Geometry Optimized [Tp$^{Bu^t,Me}$]MgX (X = F, Cl, Br, I).

[Tp$^{Bu^t,Me}$]MgF

atom	x	y	z
Mg	4.0428056014	4.2993846538	4.8384416525
F	5.7534021063	4.7818972314	4.6507477707
B	1.1605139171	3.4645364176	5.1673298384
H	0.0202166729	3.1353301808	5.2980455519
N	1.3611225168	4.8738436827	5.7978607770
N	2.5889697692	5.4848300349	5.7765546539
N	2.0769620178	2.4293416970	5.8829370827
N	3.4405172738	2.5814993455	5.8802988340
N	1.5094607975	3.5086284322	3.6505740785
N	2.7663587624	3.8641411745	3.2306748626
C	-0.9776673923	5.3256252740	6.6161523878
H	-1.4937467397	5.1626254113	5.6644024175
H	-1.4837177704	6.1397640117	7.1404781566
H	-1.0951531098	4.4141700786	7.2112279097
C	0.4638022314	5.6801422127	6.4164164879
C	1.1328497670	6.8362711704	6.8011471029
H	0.7005383051	7.6786388009	7.3179759913
C	2.4653971375	6.6759455504	6.3814091742
C	3.6439084810	7.6286785551	6.5332518601
C	3.1851013722	8.9002941534	7.2733961582
H	2.8073152458	8.6690679728	8.2751031364
H	2.3968076398	9.4247061477	6.7226803294
H	4.0283397451	9.5892467995	7.3855724952

C	4.7782820722	6.9529473138	7.3404028657
H	4.4136696701	6.5750668801	8.3011773586
H	5.5742045597	7.6789581725	7.5412851353
H	5.2295033343	6.1279491958	6.7811934433
C	4.1928489842	8.0229927091	5.1411716557
H	4.6548878429	7.1682470817	4.6384743942
H	4.9679562596	8.7898591026	5.2505424447
H	3.4025166253	8.4276939602	4.5005512406
C	0.3420943058	0.8195093831	6.7455794746
H	-0.1577358376	0.6222197502	5.7916043047
H	-0.2662100362	1.5506091926	7.2878066002
H	0.3491024371	-0.1089007453	7.3216889225
C	1.7483801685	1.2965699392	6.5510798685
C	2.9296065783	0.7088603348	6.9878301950
H	3.0138453091	-0.2064261072	7.5515479594
C	3.9707349902	1.5447077863	6.5468531492
C	5.4737993203	1.3904030781	6.7416954607
C	6.1884396331	1.3113028095	5.3712862293
H	5.7565753295	0.5274078746	4.7404576704
H	7.2494770449	1.0806114536	5.5197647844
H	6.1387526731	2.2660254932	4.8395650735
C	6.0376005235	2.6001593747	7.5246482396
H	5.5027791002	2.7490533191	8.4683868423
H	5.9789845777	3.5186228793	6.9333432970
H	7.0954746420	2.4323911210	7.7563971981
C	5.7580204195	0.0989529879	7.5333211356
H	5.2896867899	0.1211796258	8.5231982360
H	6.8368464007	-0.0183766026	7.6774599926

H	5.3941295420	-0.7871392067	7.0021178218
C	-0.6946774861	2.7890718339	2.6647279861
H	-0.8065534772	1.8582543012	3.2303007492
H	-1.0887418980	2.6230422707	1.6591953087
H	-1.3182859158	3.5449073799	3.1529926647
C	0.7372763042	3.2188995374	2.5744053567
C	1.5177176572	3.3945458552	1.4377936948
H	1.1992440902	3.2445072310	0.4185508110
C	2.7855186801	3.7999123851	1.8908250327
C	4.0312450389	4.1376784620	1.0818913692
C	4.4381138364	5.6109997692	1.3226909602
H	3.6078037557	6.2933922377	1.1136349988
H	5.2726786175	5.8792272574	0.6650868649
H	4.7746360566	5.7675233146	2.3516388527
C	5.2084715582	3.2233003732	1.4975701425
H	5.5495254873	3.4458634650	2.5129393250
H	6.0605373719	3.3897913597	0.8287524201
H	4.9324455243	2.1654542531	1.4368662431
C	3.7405277726	3.9340766007	-0.4178526411
H	2.9299522212	4.5844049758	-0.7639351401
H	3.4648643267	2.8970326975	-0.6373527505
H	4.6331576464	4.1734800644	-1.0046311592

[Tp$^{Bu^t,Me}$]MgCl

atom	x	y	z
Mg	4.0483036928	4.2949111180	4.8192670317
Cl	6.2205890819	4.9349328332	4.5664341056
B	1.1805915264	3.4581916048	5.1501158665

H	0.0424113348	3.1265317772	5.2809255925
N	1.3672982657	4.8637202973	5.7844288619
N	2.5874011260	5.4988343113	5.7798957643
N	2.0884756557	2.4234264219	5.8687474785
N	3.4573236100	2.5557320315	5.8832162731
N	1.5165187288	3.5043849093	3.6348737026
N	2.7678462400	3.8558343615	3.1854457371
C	-0.9881578661	5.2593249482	6.5907222509
H	-1.4949231983	5.0961872727	5.6341635853
H	-1.5108818007	6.0608430698	7.1181667037
H	-1.0946038727	4.3415302624	7.1779441094
C	0.4480870582	5.6414755420	6.4053610231
C	1.0881565565	6.8044829616	6.8110512929
H	0.6336750217	7.6302520713	7.3348949353
C	2.4266000532	6.6813874401	6.4032237306
C	3.5585509146	7.6809451378	6.6010543145
C	3.0289098439	8.9200363255	7.3511618978
H	2.6389193224	8.6579836107	8.3404143522
H	2.2343368588	9.4237283168	6.7905176697
H	3.8422366460	9.6387282771	7.4939966767
C	4.6906831380	7.0453468730	7.4406554131
H	4.3124573115	6.6943149351	8.4066253756
H	5.4749303981	7.7862397089	7.6318083470
H	5.1594632569	6.2052784886	6.9224716885
C	4.1133469774	8.1353831655	5.2313184475
H	4.5705224751	7.3083204031	4.6826807365
H	4.8885039415	8.8961222502	5.3759040833
H	3.3212848917	8.5706283417	4.6127582734

C	0.3111454703	0.8630973021	6.7406681189
H	-0.1840238561	0.6646307309	5.7847233775
H	-0.2883999829	1.6102557280	7.2705694397
H	0.2975523315	-0.0589992943	7.3267074988
C	1.7278218510	1.3122112185	6.5545550731
C	2.8893955255	0.7129085016	7.0222878707
H	2.9470289888	-0.1920986711	7.6059065572
C	3.9533383683	1.5185378461	6.5836946786
C	5.4428460677	1.3121041273	6.8251322120
C	6.1834512140	1.1478309084	5.4778916568
H	5.7684040596	0.3142156676	4.9015186903
H	7.2444788601	0.9418409016	5.6575170089
H	6.1287601157	2.0543448132	4.8702512526
C	6.0287956669	2.5137298658	7.6020143428
H	5.5016124657	2.6621592561	8.5503962418
H	5.9738776999	3.4387974277	7.0230472762
H	7.0862694382	2.3342078622	7.8255350078
C	5.6580486974	0.0349527957	7.6622674988
H	5.1696028671	0.1052228215	8.6399593599
H	6.7284863491	-0.1157070194	7.8350602722
H	5.2753443009	-0.8539344058	7.1496020550
C	-0.7216742347	2.8112932726	2.7052804304
H	-0.8293328882	1.8810368964	3.2722936873
H	-1.1399542220	2.6506134024	1.7087502406
H	-1.3267166467	3.5725501325	3.2081729509
C	0.7115687008	3.2280364760	2.5808423506
C	1.4594443898	3.4049606497	1.4246999809
H	1.1101277623	3.2643506009	0.4141865165

C	2.7422519497	3.7983351410	1.8406601244
C	3.9490088031	4.1211595203	0.9694089759
C	4.3949274690	5.5839883441	1.1975055086
H	3.5748531702	6.2801584432	0.9919548758
H	5.2258233094	5.8301449202	0.5270923128
H	4.7448065593	5.7482722609	2.2196160226
C	5.1152450645	3.1602989628	1.2965771956
H	5.4774209607	3.2926918506	2.3189118077
H	5.9595333898	3.3558472751	0.6262329395
H	4.8115472445	2.1163780943	1.1648197356
C	3.5742554403	3.9490186721	-0.5166195856
H	2.7628121005	4.6234507236	-0.8101221359
H	3.2656852908	2.9221738056	-0.7396840580
H	4.4415967917	4.1795367912	-1.1434752563

[$Tp^{Bu^t,Me}$]MgBr

**atom**	**x**	**y**	**z**
Mg	3.9868113174	4.2769616826	4.8264619429
Br	6.3402734178	4.9683988093	4.5541754832
B	1.1227250867	3.4400150700	5.1542790856
H	-0.0148406515	3.1078935990	5.2844016331
N	1.3047103873	4.8452698515	5.7874577257
N	2.5217933030	5.4883524256	5.7872989767
N	2.0286376979	2.4040560868	5.8735300822
N	3.3997092004	2.5255324554	5.8867719947
N	1.4575050387	3.4830775147	3.6389073770
N	2.7079092335	3.8336854784	3.1830828649
C	-1.0591265979	5.2267825392	6.5779397480

H	-1.5606953524	5.0610828628	5.6188631689
H	-1.5894674374	6.0249481491	7.1030917412
H	-1.1633770833	4.3081085338	7.1644661953
C	0.3758147667	5.6179946435	6.3998314750
C	1.0048994924	6.7866210993	6.8050724980
H	0.5416043982	7.6110916927	7.3234727743
C	2.3463750247	6.6730110934	6.4059836294
C	3.4632962588	7.6880112043	6.6108956751
C	2.9093851005	8.9212249997	7.3537279082
H	2.5158977917	8.6564317697	8.3410116864
H	2.1117181977	9.4116142502	6.7854537125
H	3.7111617749	9.6522289900	7.5001410636
C	4.5947579042	7.0711298150	7.4650081481
H	4.2123484585	6.7314895522	8.4336457198
H	5.3737455386	7.8191537447	7.6501691085
H	5.0716330729	6.2259176195	6.9631556877
C	4.0200183765	8.1511763934	5.2451143171
H	4.4812103873	7.3298302378	4.6917394935
H	4.7917703393	8.9144883378	5.3949396396
H	3.2267271995	8.5860749360	4.6275549722
C	0.2393427028	0.8490742568	6.7341191344
H	-0.2562449099	0.6552262089	5.7771934274
H	-0.3569294141	1.5979959249	7.2655411283
H	0.2201656833	-0.0746204808	7.3178359647
C	1.6591395687	1.2902599984	6.5504868127
C	2.8161105690	0.6773971653	7.0111346157
H	2.8673022281	-0.2330048173	7.5866463810
C	3.8868561762	1.4757507544	6.5777859263

C	5.3733112895	1.2409569484	6.8161808496
C	6.1088482752	1.0733260738	5.4667694272
H	5.6848257636	0.2433521294	4.8913134664
H	7.1691163657	0.8591179288	5.6421429313
H	6.0588956653	1.9797389982	4.8589012850
C	5.9795198010	2.4221670738	7.6078369610
H	5.4626894288	2.5576251672	8.5640603460
H	5.9273964737	3.3588391621	7.0478702236
H	7.0374711704	2.2284594119	7.8174167907
C	5.5678645784	-0.0488971914	7.6400449990
H	5.0820998275	0.0188816860	8.6194400562
H	6.6360818682	-0.2189780945	7.8097309120
H	5.1701656342	-0.9260278965	7.1183449602
C	-0.7821584502	2.7746584217	2.7215778641
H	-0.8851304024	1.8469934568	3.2940527055
H	-1.2025612840	2.6062807687	1.7269930167
H	-1.3893263683	3.5365091920	3.2213392806
C	0.6494211601	3.1960526854	2.5902726634
C	1.3925116379	3.3662125434	1.4303402566
H	1.0399898809	3.2168753167	0.4218923746
C	2.6759137427	3.7658800015	1.8375016020
C	3.8737763742	4.0808340494	0.9505257462
C	4.3188983241	5.5470490751	1.1546833419
H	3.4979567022	6.2384172340	0.9357780012
H	5.1508203559	5.7827879390	0.4815554377
H	4.6664593279	5.7306540161	2.1740388228
C	5.0420579555	3.1208304432	1.2728536142
H	5.4077112192	3.2502043483	2.2941951775

H	5.8845117346	3.3172677322	0.6001748139
H	4.7376698229	2.0769017602	1.1405977048
C	3.4834386236	3.8921719492	-0.5299059710
H	2.6684282271	4.5629121513	-0.8225624521
H	3.1737912419	2.8624536272	-0.7385742914
H	4.3443552445	4.1166584000	-1.1679935243

$[Tp^{Bu^t,Me}]MgI$

**atom**	**x**	**y**	**z**
Mg	3.9065557076	4.2483670180	4.8277662273
I	6.4817434786	4.9985736112	4.5201791201
B	1.0377146230	3.4096957379	5.1602636503
H	-0.0990820087	3.0774147437	5.2919315834
N	1.2166654855	4.8129650656	5.7953800399
N	2.4315554443	5.4621983292	5.8033303225
N	1.9398021128	2.3739578823	5.8792674806
N	3.3121360284	2.4897769138	5.9018122364
N	1.3652851533	3.4534198607	3.6457611319
N	2.6117364569	3.8052935437	3.1769525815
C	-1.1532685346	5.1768032062	6.5808427165
H	-1.6511680480	5.0123907439	5.6196821167
H	-1.6885612805	5.9711194668	7.1069198199
H	-1.2558017371	4.2559276128	7.1641211878
C	0.2805927567	5.5755681186	6.4092482157
C	0.9009731453	6.7443849380	6.8252752963
H	0.4307383462	7.5621183084	7.3479956017
C	2.2443964755	6.6418022454	6.4314265807
C	3.3463748370	7.6682992029	6.6562092520

C	2.7738087355	8.8851604890	7.4126352867
H	2.3766792946	8.6024599083	8.3934241690
H	1.9753001922	9.3760652638	6.8460442873
H	3.5672225156	9.6218239233	7.5749516048
C	4.4785650693	7.0553011683	7.5109941563
H	4.0929303770	6.7044902579	8.4742960947
H	5.2506473477	7.8076007718	7.7067213537
H	4.9650612541	6.2174199982	7.0067545430
C	3.9019973145	8.1585024195	5.2998809141
H	4.3678721347	7.3505207425	4.7312691616
H	4.6688384377	8.9238994282	5.4629153974
H	3.1060050956	8.5984602348	4.6893707608
C	0.1337057374	0.8361966151	6.7376633324
H	-0.3584747416	0.6431029376	5.7789125404
H	-0.4608772366	1.5895691765	7.2645993256
H	0.1060257506	-0.0860049738	7.3233247791
C	1.5572763768	1.2681078798	6.5607731382
C	2.7057051610	0.6524574768	7.0358418941
H	2.7463005056	-0.2534625930	7.6189146108
C	3.7848701981	1.4408726607	6.6069433184
C	5.2636890594	1.1918327835	6.8705494806
C	6.0172895917	0.9982369943	5.5351550895
H	5.5962365259	0.1606870191	4.9684849884
H	7.0738389688	0.7818490446	5.7283692841
H	5.9799883274	1.8929067735	4.9097008947
C	5.8691988270	2.3719486733	7.6636618336
H	5.3405567232	2.5156202740	8.6122139481
H	5.8309704434	3.3069843067	7.1004527378

H	6.9229117213	2.1722524431	7.8878275625
C	5.4303146863	-0.0917322487	7.7103705540
H	4.9295383857	-0.0087660448	8.6809222580
H	6.4937688330	-0.2706956279	7.8990416088
H	5.0323169124	-0.9701691265	7.1911870846
C	-0.8872099588	2.7500234560	2.7534055232
H	-0.9868627916	1.8234908127	3.3281096119
H	-1.3170443078	2.5811561548	1.7629829957
H	-1.4884190677	3.5137777335	3.2573994306
C	0.5437706355	3.1686970911	2.6073117088
C	1.2724184357	3.3389354507	1.4392705252
H	0.9072797447	3.1910858819	0.4353281705
C	2.5607642486	3.7366343378	1.8301833501
C	3.7402267307	4.0487477963	0.9190379669
C	4.1913670912	5.5139882548	1.1145170136
H	3.3687469703	6.2059220843	0.9036862394
H	5.0159194193	5.7470263936	0.4317026919
H	4.5499535109	5.7011646383	2.1291211331
C	4.9093759830	3.0815771345	1.2131848473
H	5.2912303560	3.1970388635	2.2299730568
H	5.7425156550	3.2807669693	0.5300821861
H	4.5973667686	2.0406350716	1.0753938103
C	3.3198554759	3.8648573110	-0.5540515512
H	2.5024476251	4.5397231645	-0.8297958743
H	3.0024277229	2.8369439738	-0.7596493855
H	4.1695199996	4.0874166292	-1.2076167290

REFERENCES

(1) (a) McNally, J. P.; Leong, V. S.; Cooper, N. J. in *Experimental Organometallic Chemistry*, Wayda, A. L.; Darensbourg, M. Y., Eds.; American Chemical Society: Washington, DC, 1987; Chapter 2, pp 6-23. (b) Burger, B. J.; Bercaw, J. E.

(b) Burger, B.J.; Bercaw, J. E. in *Experimental Organometallic Chemistry*; Wayda, A. L.; Darensbourg, M. Y., Eds.; American Chemical Society: Washington, DC, 1987; Chapter 4, pp 79-98.

(c) Shriver, D. F.; Drezdzon, M. A.; *The Manipulation of Air-Sensitive Compounds*, 2nd Edition; Wiley-Interscience: New York, 1986.

(2) Fulmer, G. R.; Miller, A. J. M.; Sherden, N. H.; Gottlieb, H. E.; Nudelman, A.; Stoltz, B. M.; Bercaw, J. E.; Goldberg, K. I. *Organometallics* **2010**, *29*, 2176-2179.

(3) (a) Harris, R. K.; Becker, E. D.; De Menezes, S. M. C.; Goodfellow, R.; Granger, P. *Pure Appl. Chem.* **2001**, *73*, 1795-1818.

(b) Harris, R. K.; Becker, E. D.; De Menezes, S. M. C.; Granger, P.; Hoffman, R. E.; Zilm, K. W. *Pure Appl. Chem.* **2008**, *80*, 59-84.

(4) Chakrabarti, N.; Sattler, W.; Parkin, G. *Polyhedron* **2013**, *58*, 235-246.

(5) Yousef, R. I.; Walfort, B.; Ruffer, T.; Wagner, C.; Schmidt, H.; Herzog, R.; Steinborn, D. *J. Organomet. Chem.* **2005**, *690*, 1178-1191.

(6) Krause, E. *Ber. Dtsch. Chem. Ges.* **1918**, *51*, 1447-1456.

(7) (a) Seyferth, D.; Kahlen, N. *J. Org. Chem.* **1960**, *25*, 809-812.

(b) Seyferth, D. *J. Org. Chem.* **1957**, *22*, 1599-1602.

(8) (a) Sheldrick, G. M. SHELXTL, An Integrated System for Solving, Refining, and Displaying Crystal Structures from Diffraction Data; University of Göttingen, Göttingen, Federal Republic of Germany, 1981.

(b) Sheldrick, G. M. *Acta Cryst.* **2008**, *A64*, 112-122.

(9) Jaguar 7.7, Schrödinger, LLC, New York, NY 2010.

(10) (a) Becke, A. D. *J. Chem. Phys.* **1993**, *98*, 5648-5652.

(b) Becke, A. D. *Phys. Rev. A* **1988**, *38*, 3098-3100.

(c) Lee, C. T.; Yang, W. T.; Parr, R. G. *Phys. Rev. B* **1988**, *37*, 785-789.

(d) Vosko, S. H.; Wilk, L.; Nusair, M. *Can. J. Phys.* **1980**, *58*, 1200-1211.

(e) Slater, J. C. *Quantum Theory of Molecules and Solids, Vol. 4: The Self-Consistent Field for Molecules and Solids*; McGraw-Hill: New York, 1974.

(11) (a) Hay, P. J.; Wadt, W. R. *J. Chem. Phys.* **1985**, *82*, 270-283.

(b) Wadt, W. R.; Hay, P. J. *J. Chem. Phys.* **1985**, *82*, 284-298.

(c) Hay, P. J.; Wadt, W. R. *J. Chem. Phys.* **1985**, *82*, 299-310.

(12) Michel, O.; Dietrich, H. M.; Litlabø, R.; Törnroos, K. W.; Maichle-Mössmer, C.; Anwander, R. *Organometallics* **2012**, *31*, 3119-3127.

(13) Bhadury, P. S.; Pandey, M.; Jaiswal, D. K. *J. Fluorine Chem.* **1995**, *73*, 185-187.

(14) Hynes, M. J. *J. Chem. Soc., Dalton Trans.* **1993**, 311-312.
